# Thinking outside the ORF: UTR editing enables the functional characterization of essential ribosomal protein

**DOI:** 10.1093/plphys/kiag051

**Published:** 2026-02-27

**Authors:** Alyssa Kearly

**Affiliations:** Assistant Features Editor, Plant Physiology, American Society of Plant Biologists, Rockville, MD, United States; Boyce Thompson Institute, Cornell University, Ithaca, NY, United States

The translation of messages encoded in RNA molecules is the final step in the flow of genetic information from protein-coding genes to functional proteins. Essential to the process of translation are ribosomes, complex macromolecular machines composed of ribosomal RNAs (rRNAs) and proteins that work together to interpret the RNA message and synthesize the encoded protein. Ribosomal proteins are highly conserved across the tree of life and play critical roles in the preprocessing of rRNAs, ribosome assembly, and ribosome catalytic function. In Arabidopsis, 392 putative ribosomal proteins have been identified (Scarpin et al 2023; Lan et al 2022), including many sets of paralogous proteins. With their high levels of sequence similarity, paralogs can occupy similar functional niches within the ribosome, though often in distinct subcellular, tissue, or environmental contexts.

Many ribosomal proteins remain functionally uncharacterized due to challenges imposed by their essential nature (Meinke 2020). Classic genetics methods like generating null allele mutants cannot be applied to crucial genes as the loss of their expression is lethal, requiring researchers to take unconventional, innovative approaches to investigate essential gene function. In a study recently published in *Plant Physiology*, Chen et al (2026) exploited the regulatory capacity of the mRNA 5′ untranslated region (5′ UTR) to develop viable Arabidopsis mutants with reduced expression of the essential ribosomal protein UL3Z, and employed these mutants to explore the functional distinction between UL3 paralogs (Fig. 1).

**Figure 1:**
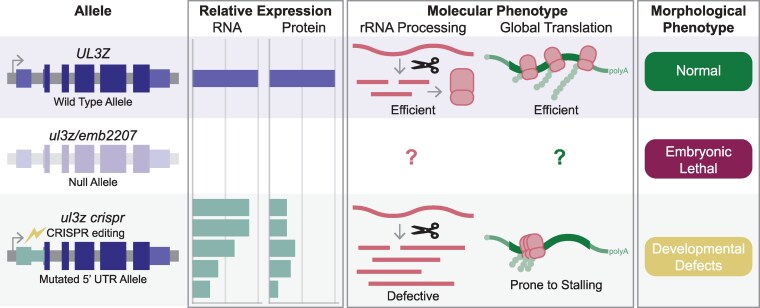
Hypomorphic alleles of *UL3Z* enabled the characterization of the UL3Z ribosomal protein. To characterize the UL3Z ribosomal protein, Chen et al. (2026) edited the 5′ UTR of the *UL3Z* gene to generate mutants harboring hypomorphic alleles. Expression of UL3Z mRNA and protein was reduced in these mutants compared to wild type, but sufficient to avoid the embryonic lethality observed in null allele mutants. At the molecular level, the *ul3z crispr* mutants displayed defects in rRNA maturation, accumulating processing intermediates. The ribosomes that were successfully assembled were more prone to stalling during translation, disrupting global translation dynamics. These issues likely contribute to the developmental defects observed at the morphological level in the *ul3z crispr* mutants.

The ribosomal protein UL3 (previously known as RPL3 in now-outdated nomenclature) is a conserved component of the large ribosomal subunit that is located in, and indeed essential for the activity of, the peptidyltransferase center of the ribosome (Schulze and Nierhaus 1982; Herold and Nierhaus 1987). However, the precise molecular function of UL3 within the ribosome, along with any functions it may have beyond translation, remain a mystery. The Arabidopsis genome encodes two UL3 proteins that associate with cytosolic ribosomes, UL3Z and UL3Y (formerly RPL3A/EMB2207 and RPL3B, respectively). Importantly, the deletion of UL3Z results in embryonic lethality (Tzafrir et al 2004), precluding the gene from being studied with traditional knockout methods. Therefore, instead of eliminating gene expression completely, Chen et al (2026) aimed to reduce expression of UL3Z to levels that result in a visible but survivable phenotype, specifically by targeting the 5′ UTR.

The 5′ UTRs of mRNAs have important regulatory functions, impacting mRNA stability and translation capacity through, for examples, the binding of RNA-binding proteins and the presence of upstream open reading frames (Hardy and Balcerowicz 2024). Using CRISPR gene editing, Chen et al (2026) generated five mutant lines with different 5′ UTR disruptions that lowered UL3Z expression to varying degrees. The *ulz3* mutant seedlings displayed a litany of developmental and reproductive defects that increased in severity with decreasing UL3Z expression. In contrast, *ul3y* TDNA mutants showed no discernible phenotype despite ablated UL3Y expression. Fascinatingly, the *ul3z* phenotypes were rescued via complementation with not only wild type UL3Z, but also with UL3Y when expressed from the *UL3Z* promoter. This finding suggests that the paralogs have redundant roles but may have distinct expression patterns that prevent endogenous UL3Y from compensating for the loss of UL3Z, even though UL3Z *can* compensate for the loss of UL3Y.

To follow up on these genetic findings, the authors performed reporter assays to determine if the UL3 paralogs display distinct expression or subcellular localization patterns that could explain the unilateral compensatory capacity. The *UL3Z* promoter showed strong, ubiquitous activity, in sharp contrast to the activity of the *UL3Y* promoter, which was markedly weaker and restricted mainly to leaves. Subcellularly, both paralogs were detected in the nucleus and the cytosol, and both were incorporated into functional ribosomes. As such, although both proteins function similarly, the loss of UL3Z has a more significant impact on plant development due to its ubiquitous expression, and UL3Y cannot compensate for that loss due to its tissue-restricted expression.

Having observed the impacts of UL3 proteins loss at the morphological level, the authors next sought to explore their roles at the molecular level. As UL3 proteins are present in the nucleus, it is possible that they are involved in pre-rRNA processing. Indeed, *ulz3* mutants were found to accumulate several rRNA processing intermediates from all three pathways of rRNA maturation, suggesting a defect early in the process. Interestingly, the accumulated intermediates frequently had non-encoded 3′ tails, which the authors hypothesize could be marking the transcripts for degradation. Additionally, *ul3* mutants displayed defects in translation. Ribosome profiling was used to measure global translation efficiency by comparing the ratios of polysomes, indicative of high translational activity, to monosomes. While it originally appeared that *ul3z* mutants had a higher proportion of polysomes to monosomes, digestion with RNase to release individual monosomes from the polysomes resulted in higher proportions of disomes and trisomes, reflective of translational stalling. Therefore, not only do UL3 proteins play a role in rRNA maturation, but they are also important for translational integrity.

In such an integral process as translation, perturbations can have major consequences, as observed with the embryonic lethality of *ul3z* null allele mutants. However, by targeting the 5′ UTR of the *UL3Z* gene, Chen et al. generated hypomorphic alleles that allowed for mutant characterization and exploration of the molecular functions of the UL3 proteins (Fig. 1). Mechanistic questions remain regarding how these 5′ UTR mutations lead to the decrease in UL3Z expression—whether the sequences changes are impacting regulatory ribosomal interactions with uORFs or the binding of RNA-binding proteins. It also remains unclear precisely how the defects in rRNA processing and translation efficiency impart the developmental defects observed in the mutants described in this work. With techniques like Ribo-seq becoming more mainstream, insights into these questions may be gained through the transcriptome-wide examination of mRNA-ribosome interactions. With these fresh insights and more forthcoming, our ability to interrogate the myriad essential ribosomal constituents improves and our understanding of the ribosome as an intricate macromolecular complex deepens.

## Data Availability

No new data was generated for this publication.

## References

[kiag051-B1] Chen N et al 2026. 5′UTR editing of ribosomal protein UL3Z gene unveils its critical roles in pre-rRNA processing and global mRNA translation dynamics. Plant Physiol. kiag073. 10.1093/plphys/kiag073. 41721525

[kiag051-B2] Hardy EC, Balcerowicz M. 2024. Untranslated yet indispensable-UTRs act as key regulators in the environmental control of gene expression. J Exp Bot. 75:4314–4331. 10.1093/jxb/erae073. 38394144 PMC11263492

[kiag051-B3] Herold M, Nierhaus KH. 1987. Incorporation of six additional proteins to complete the assembly map of the 50 S subunit from Escherichia coli ribosomes. J Biol Chem. 262:8826–8833. 10.1016/s0021-9258(18)47489-3. 3298242

[kiag051-B4] Lan T, Xiong W, Chen X, Mo B, Tang G. 2022. Plant cytoplasmic ribosomal proteins: an update on classification, nomenclature, evolution and resources. Plant J. 110:292–318. 10.1111/tpj.15667. 35000252

[kiag051-B5] Meinke DW . 2020. Genome-wide identification of EMBRYO-DEFECTIVE (EMB) genes required for growth and development in Arabidopsis. New Phytol. 226:306–325. 10.1111/nph.16071. 31334862

[kiag051-B6] Scarpin MR et al 2023. An updated nomenclature for plant ribosomal protein genes. Plant Cell. 35:640–643. 10.1093/plcell/koac333. 36423343 PMC9940865

[kiag051-B7] Schulze H, Nierhaus KH. 1982. Minimal set of ribosomal components for reconstitution of the 110 peptidyltransferase activity. EMBO J. 1:609–613. 10.1002/j.1460-2075.1982.tb01216.x. 6765232 PMC553095

[kiag051-B8] Tzafrir I et al 2004. Identification of genes required for embryo development in Arabidopsis. Plant Physiol. 135:1206–1220. 10.1104/pp.104.045179. 15266054 PMC519041

